# Reduced CD4 T cell activation and in vitro susceptibility to HIV-1 infection in exposed uninfected Central Africans

**DOI:** 10.1186/1742-4690-3-35

**Published:** 2006-06-22

**Authors:** Evélyne Bégaud, Loïc Chartier, Valéry Marechal, Julienne Ipero, Josianne Léal, Pierre Versmisse, Guillaume Breton, Arnaud Fontanet, Corinne Capoulade-Metay, Hervé Fleury, Françoise Barré-Sinoussi, Daniel Scott-Algara, Gianfranco Pancino

**Affiliations:** 1 Institut Pasteur, Bangui, CAR; 2 Unité de Recherche et d'Expertise Epidémiologie des Maladies Emergentes, Institut Pasteur, Paris, France; 3 Unité de Régulation des Infections Rétrovirales, Institut Pasteur, Paris, France; 4 Service de Médecine Interne, Groupe Hospitalier Pitié-Salpétrière, Paris, France; 5 INSERM, Unité 543, Groupe Hospitalier Pitié-Salpêtrière, Paris, France; 6 Université Bordeaux 2, Bordeaux, France; 7 Centre de Ressources Biologiques de l'Institut Pasteur, Institut Pasteur, Paris, France; 8 Unité Postulante Interactions Moléculaires Flavivirus-Hôtes

## Abstract

**Background:**

Environmentally driven immune activation was suggested to contribute to high rates of HIV-1 infection in Africa. We report here a study of immune activation markers and susceptibility to HIV-1 infection in vitro of forty-five highly exposed uninfected partners (EUs) of HIV-1 infected individuals in Central African Republic, in comparison with forty-four low-risk blood donors (UCs).

**Results:**

Analysis of T lymphocyte subsets and activation markers in whole blood showed that the absolute values and the percentage of HLA-DR^+^CD4 T cells and of CCR5^+^CD4 T cells were lower in the EUs than in the UCs (p = 0.0001). Mutations in the CCR5 coding region were not found in either group. Susceptibility to in vitro infection of unstimulated peripheral blood mononuclear cells, prior of PHA activation, was decreased in EUs compared to UCs, either using a CXCR4-tropic or a CCR5-tropic HIV-1 strain (p = 0.02 and p = 0.05, respectively). Levels of MIP-1β, but not of MIP-1α or RANTES, in the supernatants of PHA-activated PBMC, were higher in the EUs than in the UCs (p = 0.007).

**Conclusion:**

We found low levels of CD4 T cell activation and reduced PBMC susceptibility to HIV-1 infection in Central African EUs, indicating that both may contribute to the resistance to HIV-1 infection.

## Background

Central African Republic (CAR) has a high prevalence rate of HIV-1 infection estimated at 18% among pregnant women [1]. The primary route of transmission is heterosexual. High rates of infection in HIV-1-infected individuals in Africa have been suggested to be related to immune hyperactivation driven by environmental factors, including high exposure to infectious agents and poor hygienic conditions [2]. Indeed, higher levels of CD4 and CD8 T cell activation have been reported in HIV-1 infected Africans in comparison with European populations [3]. In particular, HLA-DR expression on CD4 T cells was correlated with CD4 T cell count, viral load and coinfections [4]. Successful antiretroviral therapy was reported to decrease the levels of T cell activation markers, with a stronger effect on CD8 than on CD4 T cell activation [4,5]. A pattern of immune activation, including an increase of activated T cell subsets and of the HIV-1 CCR5 co-receptor expression, has been reported not only in HIV-infected but also in HIV-uninfected African populations [6-9]. Interestingly, peripheral blood mononuclear cells (PBMC) from individuals with a chronic immune activation exhibited higher susceptibility to HIV-1 infection in vitro [10].

In spite of the spreading of HIV-epidemic in CAR, a consistent proportion of Central Africans who have been persistently exposed for several years to infection through unprotected sexual relationships with HIV-1 infected partners remained seronegative (Bégaud E. et al., unpublished data). Most studies on correlates of protection against HIV-1 infection in seronegative exposed individuals in other African countries were conducted on cohorts of commercial sex workers (CSW) whereas only a few studies concerned seronegative partners in serodiscordants couples [11-18]. In these studies, HIV-1-specific helper and cytotoxic T cell responses have been detected in a significant proportion of exposed seronegative individuals [19-23]. HIV-1-specific mucosal IgA were also detected in persistently negative Kenyan CSW and were shown to be capable of in vitro neutralization of HIV-1 [24,25]. The question of whether these specific immune responses exert a protective role or they reflect exposure to HIV-1 is, however, still debated [26-28]. Other immunological or genetic factors potentially related to the resistance have also been addressed in exposed seronegative African individuals but no clear protective mechanisms emerged from these studies [12,13,29-31]. Genetic polymorphism in the CCR5 gene, such as the CCR5-Δ32 mutation, which was associated to the resistance to HIV-1 infection in Caucasian populations, has not been found in Africans [32].

We studied a group of exposed seronegative partners of HIV-1 infected individuals in Bangui with a long history of leading a common life and practicing unprotected sexual relations. Considering the background of immune activation reported in African populations, we asked whether differences in the levels of CD4 T cell activation and in the capacity to replicate HIV-1 in vitro could be related to the apparent resistance to infection in this group. From the studies conducted, we found lower levels of CD4 T cell activation and reduced in vitro susceptibility to HIV-1 infection in exposed seronegative individuals than in low-risk Central African blood donors.

## Results

### Characteristics of study population

Forty-five EU partners of HIV-1 infected individuals were included in the study. Median common life with unprotected sex relations was estimated to be 8 years (Table 1). At the time of enrollment, most HIV-1 infected partners were consulting for clinical symptoms related to HIV-1 infection. Median CD4 T cell count of HIV-1 infected partners was 73 cells/μl (IQR: 26-227) and median plasma viral load was 7.2 × 10^4 ^copies/ml (IQR: 1.3 × 10^4 ^– 14.8 × 10^4^). Altogether, the clinical and virological characteristics of HIV-1 infected individuals suggested a long-term infection and a history of high risk exposure for their partners. There were no significant differences in potential risk factors for infection between the serodiscordant couples and 243 couples consulting at the Communitarian Hospital of Bangui in which both partners were seropositive (not shown). Among UCs, 73% were men and 27% women, with a median age of 24 years (18–40).

**Table 1:** Characteristics of the EUs n = 45

**Sex**:	
Male	45 %
Female	55 %
**Age **(years):	35 (18–52)*
**Common life **(years)**:**	8 (2–27)*
**Sexual intercourses/week :**	2 (1–5)*
**Never using condom :**	84.2 %
**STI antecedents:**	5.6 %
**Circumcised men:**	81.6 %

*Median (min-max)

The analysis of the CCR5 coding region polymorphism did not reveal the presence of variants of the CCR5 co-receptor, including the CCR5Δ32 mutation, among EUs or UCs.

### Decreased expression of HLA-DR and CCR5 on CD4 T cells from EUs

In order to evaluate a potential role of immune activation and co-receptor expression in the susceptibility to HIV-1 infection, we examined lymphocyte subsets, expression of activation markers (HLA-DR and CD38) and HIV-1 co-receptors (CXCR4 and CCR5) on T cells in fresh whole blood from 25 EUs and 24 UCs. We did not find differences in CD4 or CD8 T cells, B and NK cells (absolute values and percentages) between the two groups (Table 2). However, the absolute value and the percentage of HLA-DR^+^CD4 T cells were significantly lower in the EUs than in the UCs (p = 0.0001) (Table 2). We also found lower absolute value and percentage of CCR5-expressing CD4 T cells in the EUs than in the UCs (p = 0.0001) (Table 2). CCR5 and HLA-DR expression on CD4 T cells were highly correlated (r = 0.80, p < 0.0001). Conversely, the absolute value and the percentage of CXCR4-expressing CD4 T cells did not differ between EUs and UCs. Furthermore, no differences were found in the expression of activation markers or of CXCR4 and CCR5 on CD8 T cells (Table 2).

**Table 2:** Lymphocyte subsets and activation markers in the EUs and UCs

**Lymphocyte subsets**	**EUs (n = 25)**	**UCs (n = 24)**	**p-value°**
Lymphocytes *	2500 (2052–3031)	2184 (1800–2632)	0.26
CD3 (%)*	72 (69–77)	73 (70–80)	0.27
CD4 (%)*	42 (32–46)	42 (34–45)	0.98
CD8 (%)*	28 (23–33)	28 (25–33)	0.63
NK (%)*	17 (13–22)	14 (11–18)	0.19

**T cell markers** **(T cell subset)**^#^			
HLA-DR (CD4^+^)*	138.6 (61–208)	288.7 (217–403)	**0.0001**
CD38 (CD4^+^)*	527.6 (267 – 862)	269.5 (238–410)	0.13
HLA-DR (CD8^+^)*	200 (124–285)	280.1 (165–360)	0.13
CD38 (CD8^+^)*	370 (194–527)	312.3 (244–441)	0.87
CXCR4 (CD4^+^)*	1041 (475–1233)	579.2 (221–895)	0.11
CCR5 (CD4^+^)*	44.1 (26.9–88.7)	146.5 (92.4–312)	**0.0001**
CXCR4 (CD8^+^)*	470.2 (298–747)	323.2 (155–500)	0.17
CCR5(CD8^+^)*	250.5 (164–293)	169.3 (106–245)	0.20

* Median (Q1–Q3)

° Mann-Whitney U test. After Bonferroni correction the level of significance was set at 0.004

^# ^Absolute values

### Reduced PBMC susceptibility to in vitro HIV-1 infection in EUs

**Figure 1:**
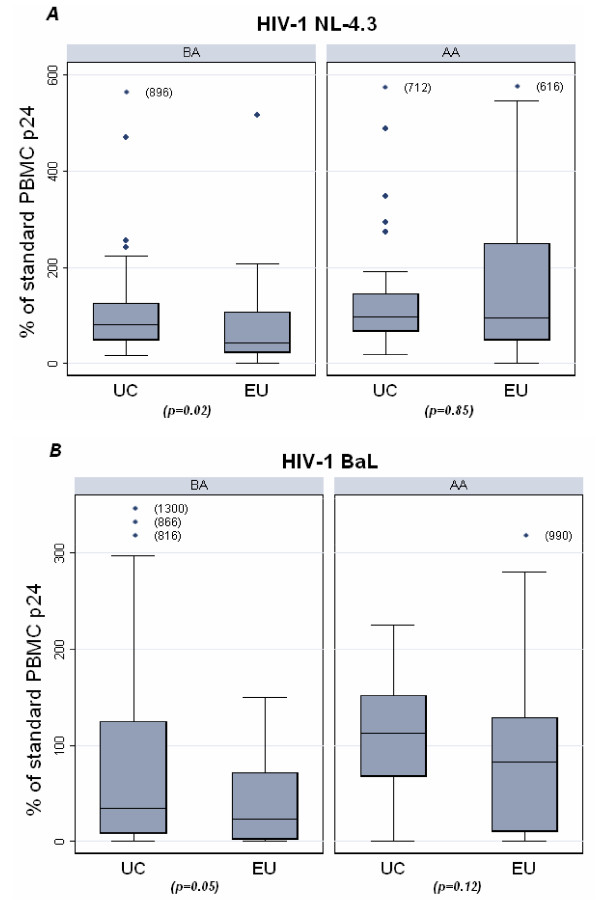
**Susceptibility of peripheral blood mononuclear cells to in vitro HIV-1 infection**. PBMCs (10^5 ^cells/well in 96-well microplates) from 45 EUs and 44 UCs were infected with HIV-1 NL-4.3 (A) or with HIV-1 BaL (B) in quadruplicate. Infections were performed either 24 h before PHA activation of PBMC (BA), or after 3-days PHA activation (AA). Standard PBMC were infected in parallel in each assay. HIV-1 replication was monitored by p24 antigen measure in culture supernatants. The p24 values of each individual PBMC infection were compared to the mean p24 value of standard PBMC. Results are expressed as the percent of the standard PBMC supernatant p24 at the peak of infection. Box-plots represent the 25^th ^and the 75^th ^percentiles and bars indicate the lowest and the highest values. The horizontal line in the boxes indicates the median. Comparisons between the groups were made using the non parametric Mann-Whitney U test.

We evaluated PBMC susceptibility to in vitro infection using two HIV-1 strains with different tropisms, HIV-1 NL-4.3 (X4) and HIV-1 BaL (R5). In order to evaluate the impact of the baseline activation of PBMC on their susceptibility to infection, we inoculated PBMC with the virus without any previous exogenous stimulation (infection before activation, BA) and then activated PBMC with PHA to allow efficient viral replication. In parallel, we also infected 3-days PHA activated PBMC (infection after activation, AA). The levels of p24 produced in infected cultures were expressed as percentages of those produced in parallel infections of standard PBMC (Fig. 1). In BA infectivity assays, we found lower p24 levels in PBMC from EUs than in PBMC from UCs after infection with either NL-4.3 (*p *= 0.02) or HIV-1 BaL (*p *= 0.05) (Fig 1A,B). In AA infectivity assays, no significant differences between EU and UC were found for both NL-4.3 and BaL infections, although p24 production in BaL infected PBMC were lower in the EUs than in the UCs (medians: 87 and 113 in EUs and UCs respectively, p = 0.12). We also performed infectivity assays using one CAR primary HIV-1 isolate on PBMC from 30 EUs and 35 UCs. BA infectivity assays with this CAR primary HIV-1 isolate indicate a lower virus production, evaluated by p24 levels in PBMC supernatants, in EUs than in UCs, as observed with BaL and NL-4.3 viruses. However, the difference did not reach statistical significance, because of the lower number of subjects analyzed (percentages of infection were 26 [0–124] and 49 [4.3–327] in EUs and UCs respectively (p = 0.14). In AA infectivity assays with the primary isolate, percentages of infection were 76 [7.9–392] and 117.3 [9.1–449.5] in EUs and UCs respectively.

### β-chemokine secretion by PBMC

We found a higher level of MIP-1β/CCL4 production in PHA-activated PBMC supernatants from the EUs than from the UCs (medians: 43.7 and 28.9 ng/ml in EUs and UCs respectively, p = 0.007), whereas there were no significant differences in MIP-1α/CCL3 (medians: 20.9 and 19.2 in EUs and UCs) or RANTES/CCL5 (medians: 30.8 and 22.4 in EUs and UCs) between the two groups.

## Discussion

Immune activation has been suggested to be critical in HIV-1 transmission and spreading in sub-Saharan Africa. We studied a group of Central African individuals who remained apparently uninfected despite repeated unprotected sex intercourses with their HIV-1-infected partners (EUs). We found a lower number and proportion of activation marker-expressing CD4 T lymphocytes in the EUs than in the low-risk controls. HLA-DR^+^CD4 T cell levels in UCs were indeed comparable to those reported for other African populations [13]. These data suggest lower availability of activated CD4 T cells for efficient HIV-1 replication in Central African EUs. Accordingly, PBMC from the EUs were less susceptible to both X4 and R5 HIV-1 strains in comparison with PBMC from the UCs, when infected before PHA-stimulation. In this condition, differences in target cell basal activation between the EUs and the UCs may eventually result in different capacity to support viral infection and replication. A higher number of activated CD4 T cells in UCs may allow a more rapid onset of HIV-1 replication, which would then promote a more efficient spread of infection upon PHA-activation. The loss of difference in HIV-1 replication levels between EUs and UCs when PBMC were activated before infection may be due to the strong polyclonal stimulation by PHA that may mask the initial differences in the susceptibility to HIV-1 infection. Still, the levels of HIV-1 Bal production were lower in the EUs PBMC, possibly due to higher production of MIP-1β. Contrasting results have been reported in this regard, depicting either lower or comparable susceptibility to HIV-1 infection of PHA-stimulated PBMC in different EU groups in comparison to control groups [12,30,33-36]. However, whether discrepancies are linked to differences in experimental conditions or to differences between populations of study remains unclear

Consistent with the low CD4 T cell activation, EUs also showed a significantly lower expression of CCR5 on CD4 T lymphocytes. CCR5 surface expression has been shown to influence in vitro infectability with R5 HIV-1 [37-41] and R5 variants are largely prevalent in infected individuals in Bangui [42]. Therefore, low levels of CCR5-expressing CD4 T cells may actually contribute to protect Central African EUs from HIV-1 transmission.

A recent study has also associated lower levels of CD4 T cell activation with resistance to HIV-1 in a group of homosexual EUs from the Amsterdam Cohort Studies [43]. In this study, lower percentages of CXCR4 – but not of CCR5-expressing CD4 T cells were found in EUs. However, lymphocyte phenotypes were determined on cryopreserved PBMCs and not in fresh blood, as we did, and the surface expression of CXCR4 and CCR5 may vary according to the nature and the conservation of samples [44] and Scott-Algara D. et al. unpublished data). A CXCR4 decreased expression in CD4 T cells from commercial sex workers in Côte d'Ivoire was associated with prolonged duration of commercial sex work and not with T cell activation [13]. It is possible that parameters associated with different sexual behaviours and/or environments are involved in the regulation of chemokine receptors expression.

## Conclusion

In summary, we found low CD4 T cell activation and CCR5 expression as well as a reduced susceptibility to HIV-1 infection in Central African EU partners of HIV-1 infected individuals. This study did not allow further examination of immune activation or HIV-specific responses in other lymphoid compartments, including genital mucosa that is particularly relevant in sexual transmission of HIV-1. Previous studies of parameters of protection in EU CSW in Kenya revealed higher frequencies of CTL, CD4 T cells, IgA or elevated levels of β-chemokines in cervico-vaginal samples than in blood [16,21,24]. However, low levels of systemic CD4 T cell activation as well as a reduced susceptibility to HIV-1 infection documented here may reflect a general lower permissiveness to infection in EUs and may contribute to the protection against HIV-1 transmission, possibly together with other anti-viral responses not addressed here. Indeed, EUs are heterogeneous populations and different mechanisms, either innate or acquired, likely account for protection in different individuals [45-47].

## Materials and methods

### Study population

Forty-five HIV-1-positive individuals and their HIV-1-negative heterosexual partners having a history of regular unprotected sexual relations for more than two years were enrolled at the Communitarian Hospital in Bangui. Seropositive patients were admitted in the hospital for medical care. Plasma HIV-1 RNA levels were quantified using the HIV-1 RNA 3.0 assay (Chiron, France). Partners of HIV-1 infected patients were tested negative for HIV-1 infection by Genelavia Mixt (Sanofi Diagnostics Pasteur) and Vironostica HIV Uni-form II (Organon Teknika) and by PCR on PBMC DNA for *gag*, *pol*, and *env *genes and will therefore be referred to as exposed uninfected individuals (EUs). All PCR primers used allow amplification of most HIV-1 isolates of the M group, including A subtype *env *that is predominant in CAR [48]. Enrolled participants gave their informed consent, completed a questionnaire regarding the frequency of sexual relations and other risk practices and received counseling and information about HIV-1 and safe sex. Forty-four Central African HIV-1 seronegative individuals with no risk factors for HIV-1 infection, such as sex with multiple partners or intravenous drug use, were recruited in Bangui as unexposed controls (UCs). This study was approved for ethical clearance by CAR Health Ministry and Health Authorities.

The political situation in CAR, including a coup in March 2003, caused movements of population including individuals enrolled in this study. Consequently, most of the analyses described below were performed on blood samples collected only at baseline visit.

### CCR5 polymorphism analysis

CCR5 coding region polymorphism in EUs was analysed by denaturing high performance liquid chromatography and sequencing, as previously described [49]

### Flow cytometry

Immunophenotyping analyses were performed on fresh whole blood samples from EUs or UCs at the Institut Pasteur of Bangui. CD4, CD8, NK and B cell populations were studied by a three-colour flow cytometry with standard lysis procedures. Labelled cells were analysed on a FACSCalibur flow cytometre (Becton Dickinson, Paris, France) (10000 events in lymphocyte gate). Monoclonal antibodies were acquired from Becton Dickinson.

### Infectivity assays

Peripheral blood mononuclear cells (PBMCs) were prepared from blood samples collected on EDTA by centrifugation on Ficoll-Paque Plus (Amersham Bioscience, Uppsala, Sweden) and cryopreserved in fetal calf serum (FCS, Life Technologies) – 10 % dimethyl sulphoxide (Sigma, Irvine, UK). Susceptibility of PBMC to HIV-1 infection was determined by using the CCR4-tropic (X4) NL-4.3 HIV-1 or the CCR5-tropic (R5) BaL HIV-1 strains. In some experiments we also used a primary R5/(X4) isolate, HIV-1 73Mcd, from one CAR HIV-1-infected partner of an EU. To reduce inter-experimental variability bias, we included a same reference stock of PBMC from a HIV^- ^blood donor used as standard in each assay and designated thereafter "standard PBMC". Viral inoculums able to consistently infect standard PBMCs were determined in preliminary assays. For each individual, PBMCs (10^5 ^cells/well in 96-well microplates) were infected in quadruplicate either 24 h before being activated with PHA (1 μg/ml) for 3 days (infection before activation: BA), or after 3-days activation with PHA (infection after activation: AA). PBMCs were then cultured in RPMI 1640 medium containing 10% FCS, 1% penicillin-streptomycin-neomycin, 1% glutamine and 100 U.I. of IL_2 _(Proleukin; Chiron, France) and HIV-1 replication was monitored by p24 antigen quantification in culture supernatants (HIV-1 p24 Antigen Assay, Coulter, France). The p24 value of standard PBMCs at their peak of infection (day 10–13) was considered to be 100 % for each infectivity assay. The p24 values of each individual PBMC infection were compared and normalised to the mean p24 value of the standard PBMC. Infectivity assays were performed in the L3 laboratory of the Institut Pasteur of Bangui.

### β-chemokine production

β-chemokine (MIP-1α/CCL3, MIP-1β/CCL4, RANTES/CCL5) levels in PHA-activated PBMC supernatants were measured using commercial ELISA kits according to the manufacturer's instructions (Quantikine R&D Systems, Oxon, UK).

### Statistical analysis

Statistical analysis was performed by using the STATA 8.0 statistical package (Stata Corporation, College Station, Texas, USA). Continuous variables were compared between the groups using the non-parametric Mann-Whitney U test. A Bonferroni correction was applied to the level of significance of statistical tests for comparing lymphocyte phenotypes between EU and controls due to the large number of tests performed in this analysis. Based on 13 comparisons of phenotypes, the level of significance was set at 0.05/13 = 0.004. Spearman rank correlation coefficients were computed to assess the strength of the association between two continuous variables.

## Competing interests

The author(s) declare that they have no competing interests.

## Authors' contributions

EB, FB-S, DS-A and GP conceived of the study and contributed to its experimental design and coordination. GB and VM participated in the design and coordination of the study. LC and AF performed the statistical analysis. JI, JL and PV carried out the infectivity assays. CC-M performed CCR5 sequencing and analysis. HF contributed measures of plasma viral load. GP drafted the manuscript. All authors read and approved the final manuscript.

**Figure 2:**
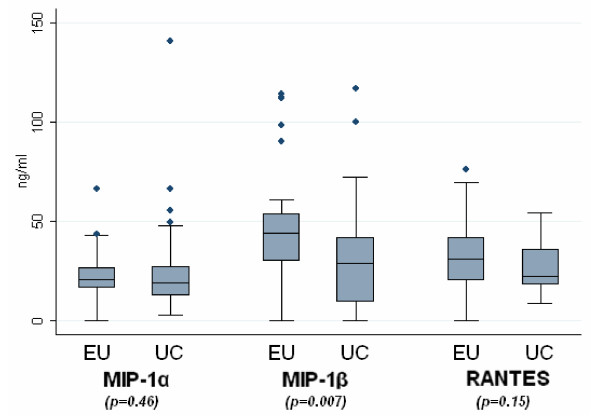
**β-chemokine levels in PBMC supernatants**. PBMC culture supernatants were collected after a 3-day-PHA-activation and levels of MIP-1α/CCL3, MIP-1β/CCL4 and RANTES/CCL5 were measured by ELISA. Box-plots show the median values and percentiles of the levels of each β-chemokine expressed in ng/ml. Comparisons between the groups were made using the Mann-Whitney U test.
